# Social network interventions in mental healthcare: a protocol for an umbrella review

**DOI:** 10.1136/bmjopen-2021-052831

**Published:** 2021-12-17

**Authors:** Marta Chmielowska, Yaara Zisman-Ilani, Rob Saunders, Stephen Pilling

**Affiliations:** 1 Centre for Outcomes Research and Effectiveness (CORE), Research Department of Clinical, Educational and Health Psychology, University College London, London, UK; 2 Social and Behavioural Sciences, Temple University College of Public Health, Philadelphia, Pennsylvania, USA

**Keywords:** mental health, psychiatry, protocols & guidelines

## Abstract

**Introduction:**

Social networks (SNs) can play a crucial role in the process of recovery from mental illness. Yet there is no standard best practice for involving SNs to optimise patient recovery. It is therefore critical to explore the diversity of SN approaches in mental health, highlight gaps in the evidence and suggest future directions for research and practice. This protocol describes the methods for an umbrella review of SN interventions for the care and/or treatment of mental illness.

**Methods and analysis:**

Nine electronic databases will be searched for the relevant journal articles: CINAHL, PubMed, Scopus, Ovid MEDLINE, Ovid EMBASE, Ovid Cochrane Library, Web of Science, Scopus and Ovid PsycINFO. We will include reviews which extracted information about the quantity, structure and quality of patient’s SNs as well as frequency of contact. The range of publication dates of the included articles will be from 2010 and 2021, as recommended by Joanna Briggs Institute guidelines. The Assessment of Multiple Systematic Reviews 2 tool and ratings of the quality of evidence will be used to assess the quality of the included reviews. The results will be presented in accordance with guidelines in the Cochrane Handbook for Systematic Reviews of Interventions and the Preferred Reporting Items for Systematic Reviews and Meta-Analyses 2020 statement. Findings will inform the development of an SN framework to guide the design and evaluation of psychosocial interventions.

**Ethics and dissemination:**

This umbrella review will involve secondary data analysis and ethical approval is not required. The target audience includes clinicians, researchers and service users, who will be reached with tailored materials through journal publications, conference presentations and social media. The presentation of the results will provide a more complete picture of relevant evidence and explicit basis from which to improve psychosocial well-being for people diagnosed with a mental illness.

**PROSPERO registration number:**

This protocol was registered with the International Prospective Register of Systematic Reviews (http:/ /www.crd.york.ac.uk/PROSPERO), registration number CRD42020192873.

**Strengths and limitations of this study**

- The first umbrella review of systematic review articles about social network (SN) approaches to care and/or treatment of mental illness.
- An umbrella review allows to summarise the evidence from multiple research syntheses into one systematic review of reviews.
- This umbrella review will be a critical first step towards developing a conceptual and theoretical framework of SNs approaches to care and/or treatment of mental illness.
- The search will be limited to English articles only and might exclude additional studies published in other languages, with a negligible impact on the effect estimates and conclusions.

## Introduction

Social networks (SNs) are a set of social relations that provide social support,^1^ connect individuals^2^ and are important for physical and mental health throughout life.^3–6^ SNs differ on a range of structural and functional components, such as network size, frequency of social contact and types of perceived and/or received social support.^7^ Reduced SNs and poor social support are associated with negative health outcomes, including development of a coronary heart disease and stroke,^8^ increased blood pressure,^9 10^ chronic pain,^11 12^ depression,^13^ anxiety,^13^ personality disorders,^14^ psychoses,^15^ eating disorders,^16^ sleep problems^17^ and suicidal behaviour.^18^ Strong SNs are linked to better health practices and psychological processes such as enhanced self-management, reduced use of health services,^19^ less hospitalisation,^20^ lower suicide risk/rates,^21^ greater community participation and improved quality of life.^22^

SNs are crucial for the social integration and recovery of people with mental illness who frequently experience difficulties in developing and maintaining social relationships and are more socially isolated and lonely compared with the general population.^15 23 24^ However, the conceptualisation and operationalisation of SNs varies across studies, limiting the comparability of their results. The terms of SNs and social support are often used interchangeably^25^ and many studies fail to distinguish between SNs and social support within their analyses.^26 27^

A wide range of SN interventions are used to help improve care and treatment of mental illness, though evidence of their effectiveness is minimal.^28 29^ Inconsistent definitions and measurement of SNs across studies results in a limited understanding of what constitutes appropriate SN involvement and how to best incorporate SNs into mental health services.^30^ Such discord is problematic and impedes the implementation and sustainability of interventions aimed at increasing social support for patients with mental illness. Recent evidence which suggests that self-imposed isolation (or self-retreat) can aid recovery from mental illness further complicates this issue.^31^ Therefore, there is an urgent need to better understand the role of SNs in patient recovery, to define what is considered an effective SN intervention and to determine the key elements and steps which are essential to its successful implementation in mental health.

This protocol describes the methods for the conduct of an umbrella review to identify and define the key components of SN interventions in mental healthcare, and to support the implementation of effective strategies to address the psychosocial needs of those with mental health problems. The aim of this exploratory review is to evaluate the strength and credibility of the evidence on best practices for involving SNs in care and treatment of mental illness.

## Objectives

1. Identify the published systematic reviews and/or meta-analyses which synthesise the available evidence on the effectiveness of SN interventions for care and/or treatment of mental illness.
2. Assess the scope and quality of the identified systematic review articles, and to provide a comprehensive evidence map of the effectiveness of SN interventions, including key elements or principles associated with better outcomes.
3. Develop a typology classification of SN approaches in mental healthcare which will be used as a guide for implementation of evidence-based practices for care and treatment of mental illness.

## Methods

### Protocol and registration

Methods for the umbrella review were developed based on criteria for conducting overviews of reviews in the Cochrane Handbook of Systematic Reviews of Interventions. The anticipated start date of this study was in July 2020 which was postponed until September 2021 due to COVID-19-related disruption. The estimated end date for this study is in November 2021.

### Ethics

Ethical approval for the conduct of this study is not required as the umbrella review will analyse previously collected data. Results will be published in a peer-reviewed journal and disseminated through conferences and/or seminars.

### Patient and public involvement

Patients and/or the public will not be directly involved in the design, or conduct, or reporting or dissemination plans of this research.

### Eligibility criteria

This umbrella overview will include systematic reviews of studies which may have compared SN interventions with non-SN interventions with a similar purpose, or with usual care. We plan to include reviews that were considered to be systematic if their authors defined a strategy to search for studies, to appraise their quality and to synthesise their findings. As described in detail previously,^32^ these may consist of reviews of randomised studies, non-randomised studies, and before-and-after studies, including qualitative and observational studies, to help understand the variation in outcomes and the mechanism by which SN interventions have an impact. The excluded articles will consist of non-systematic reviews and studies that involve primary data collection such as, randomised trials and non-randomised trials. The main focus will be on systematic reviews rather than original trials in order to summarise the widest range of relevant evidence and compare the best estimates of effectiveness of different interventions. We will include reviews regardless of the statistical significance of the reported results. In a situation where the same group of authors published more than one systematic review of the same intervention and patient population, we will select the most recent review if considered by its authors as an update of their previous review(s). If multiple reviews of the same intervention and patient population are published in a short period of time (<2 years) but with conflicting results, we will explore any potential similarities and/or differences in the full texts of the reviews and lists of included studies. Finally, we will tabulate the comparison results, including the rationale for the selection of reviews.

### Quality criteria

To ensure the identified reviews are ‘systematic’, we will check if the included studies addressed the following two items of the Assessment of Multiple Systematic Reviews (AMSTAR) 2 tool^33^: Did the review authors use a comprehensive literature search strategy (eg, were at least two databases searched)? and Did the review authors use a satisfactory technique for assessing the risk of bias (RoB) in individual studies that were included in the review (eg, allocation)? Authors of other umbrella reviews have used similar criteria^34^ or limited inclusion to only Cochrane reviews to ensure a minimum level of research quality and methodological rigour.^35 36^ Therefore, we anticipate that this approach will enhance the overall strength and credibility of the proposed umbrella review.

### Types of interventions

We will use the following definition of a SN intervention by Speck and Attneave: ‘a needs adapted intervention which combines at least two people in a service users’ SN to bring about therapeutic change.’^37^ We will also consider more recent definitions of SNs which are referred to as sets of social contacts through which a mentally ill person develops and maintains his/her personal and social identity.^38–43^ We will summarise the nature of SN interventions as well as their impact on the quantity, structure, and quality of patient’s SNs, as well as associated outcomes for persons diagnosed with a mental illness. We will use a typology by Heaney and Israel that includes four SN interventions: (1) development of new SN ties; (2) enhancement of existing SN ties; (3) enhancement of SN through the use of natural community helpers and (4) enhancement of SNs at the community level through participatory problem-solving processes.^44^ The four interventions represent strategies that can be used, singly or in combination, by practitioners to help patients restructure or reshape their networks. SN will be defined as a set of significant social relations or social ties of an individual. It may include immediate and extended family members, friends, colleagues, members of the community and healthcare professionals. We will include reviews of interventions that involved patients’ SNs (eg, families, friends, peers and communities) in any capacity (eg, self-help groups, SN therapy, skills training, support groups, and family therapy). Studies had to evaluate interventions targeting community networks (ie, network of networks like neighbourhoods, families and churches) based on enduring social relationships likely to be involved in the patients’ lives over the long periods of time required for self-management. Interventions could take place in a wide range of healthcare settings (eg, inpatient, outpatient, community based) and will not be restricted by the mode or intensity of delivery.

### Types of participants

Participants will include adults (aged 18 years and over) who have been diagnosed with a mental illness and their SN (eg, immediate and extended family members, friends, colleagues, members of the community and healthcare professionals).

A mental illness, also called a mental health disorder, will be defined as diagnosable psychological problems which can disrupt thinking, feeling, behaviour or mood, and can cause significant distress or impairment of personal functioning.^45–47^ Some of the examples include mood disorders, anxiety disorders, personality disorders, alcohol and substance use disorders, schizophrenia and psychotic disorders. We will exclude systematic reviews targeting patients with mild cognitive impairment, dementia, learning disabilities and an acquired brain injury.

### Outcome measures

We will include reviews of eligible studies which extracted any types of patient and/or observer reported outcome measures (PROMs or ObsROMs) of SNs and mental illness. PROMs are direct reports from patients regarding their health condition registered via validated questionnaires with robust psychometric properties.^48^ ObsROMs are reports made by a proxy who is in direct contact with the patient when it is not possible to obtain self-reports.^49^ Our classification of outcome measures of SN will build on a distinction frequently referred to in the literature, the difference between the functional (qualitative) and structural (quantitative) aspects of social relationships.^50^ Simultaneously studying social relationship structural measures (eg, SN size, density, composition and frequency of social interactions) and functional measures (eg, emotional, instrumental or informational support provided by members of the network) will allow us to complete a more robust assessment of SNs.^51^ This approach is meaningful because of the wide degree of variation that can exist in the intensity, frequency, extent and type of support provided throughout a SN, particularly when some ties may not be supportive at all.^8^ The mental health outcomes of interest will include intermediate outcomes (eg, symptom improvement, remission, adherence, tolerability) and long-term outcomes (length of time in remission, decreased morbidity and mortality from psychiatric diagnosis) of some of the following conditions: mood disorders, anxiety disorders, personality disorders, eating disorders, alcohol and substance use disorders, schizophrenia and psychotic disorders. Composite outcomes of two or more of these outcomes (eg, psychological impairment defined by anxiety or depression) will also be eligible.

### Search strategy for identification of relevant studies

We will search nine databases: CINAHL, PubMed, Scopus, Ovid MEDLINE, Ovid EMBASE, Ovid Cochrane Library, Web of Science, Scopus and Ovid PsycINFO. We will include systematic reviews published between 2010 and 2021 which are limited to English language only. Based on Joanna Briggs Institute guidance, review articles published in the last 10 years can provide sufficient evidence base that captures primary research conducted over the previous 30 or so years.^52^ The search strategy was initially designed for Ovid Medline (please see online supplemental appendix 1), then further adapted to other databases. Full search strategies for all databases will be publicly available after the review is completed.

10.1136/bmjopen-2021-052831.supp1 Supplementary data

### Selection of studies

The primary reviewer (MC) will perform the initial screen of titles and abstracts, with a random 10% sample screened by a secondary reviewer. Disagreements will be resolved by discussion between the reviewers, with a senior reviewer appointed as arbitrator and to make the final decision. All reviewers will then perform full text screening of any potentially relevant studies.

### Data extraction and management

Two reviewers will independently perform data extraction for each review and populate a predesigned data extraction form. Discrepancies will be resolved by discussion and reaching a consensus, and if necessary, arbitration by a senior reviewer. As described in detail previously,^32^ the data extraction form will include the following elements: an assessment of methodological quality of the included review; the objectives of the review; a summary of the included studies; the interventions studied, the control conditions (if appropriate); the outcomes and time-points assessed/evaluated and where relevant estimates of effectiveness, and precision; an assessment of the methodological quality and/or RoB of the included trials and judgements of the quality of the body of evidence. This information will be valuable in order to map and describe the existing evidence for the effectiveness of SN interventions in mental healthcare.

### Assessment of methodological quality of included reviews

We will use the AMSTAR 2 tool^33^ to provide a broad assessment of methodological quality of systematic reviews that include both randomised and non-randomised studies of healthcare interventions. The quality of each review will be reflected by an overall confidence rating which can be high, moderate, low or critically low depending on the number of critical flaws and/or non-critical weaknesses. Given that this is a new and revised AMSTAR tool, it will be preferred for use in future umbrella reviews/overviews. As described in detail previously,^32^ the quality appraisal will include: a table that provides a breakdown of how each systematic review was rated on each question of the tool, the rationale behind the assessments and an overall rating for each systematic review. We will then use the results of the quality/RoB assessments to help contextualise the umbrella review’s evidence base (eg, by assessing whether and to what extent SR methods may have affected the umbrella review’s comprehensiveness and results). The primary and a secondary reviewer will assess the quality of each individual text. Any discrepancies will be resolved through a consensus discussion.

### Assessment of the quality of the evidence in reviews

We will extract the Grading of Recommendations Assessment, Development and Evaluation (GRADE) ratings from each included systematic review. This approach provides guidance on how to assess the certainty in evidence and the strength of recommendations in healthcare. It has been adopted by a wide range of organisations such as WHO, Cochrane Collaboration, Agency for Healthcare Research and Quality (USA) and National Institute of Health and Care Excellence (UK). Similar to previous umbrella reviews/overviews, we will decide whether to downgrade or upgrade the evidence quality by using criteria specified by the GRADE working group. Any discrepancies in the ratings of the quality of evidence will be resolved through discussion, until consensus is reached.

### Data synthesis and presentation

We will use a rigorous gold-standard methodology of the Preferred Reporting Items for Systematic Reviews and Meta-Analyses (PRISMA) 2020^53^ in order to facilitate the development and reporting of this umbrella review protocol. The PRISMA 2020 guideline will help us improve the transparency, accuracy, and completeness of the protocol. We will determine the precise comparisons at the review stage as we expect the research articles to differ both in terms of their review methodology and reporting of outcomes. We will group data where possible according to the population, the type of intervention and outcome measure. We will present and discuss all important limitations within the evidence base. We will consider any possible influence of publication/small study biases on review findings. Finally, we will compile a list of recommendations based on the data synthesis from all studies.

### Subgroup analysis

Analysis of subgroups or subsets is not planned. However, if there are sufficient reviews focused on any particular subgroups of individuals, such as by diagnosis, then reporting by subgroups may be included.

## Ethics and dissemination

Involvement of SNs in mental healthcare is poorly implemented, despite its firm scientific, economic, legal and moral basis. Inconsistent definitions and measurement of SNs complicate efforts to understand what constitutes appropriate SN involvement and how to best incorporate SNs into mental health services. Thus, precision about what is being studied and how best to measure it is essential. The present protocol describes the methods and steps for an umbrella review of systematic reviews and meta-analyses of SN interventions for persons diagnosed with a mental illness. The proposed umbrella review will be the first crucial step towards addressing the absence of a synthesis of research for this context. This review will focus on the analysis of secondary data and is exempt from ethics approval. Differences between studies in terms of methodological factors or in the way the outcomes are defined and measured may be expected to lead to differences in the observed intervention effects. Meta-analysis will only be considered when a group of studies is sufficiently homogeneous in terms of participants, interventions and outcomes to provide a meaningful summary. The results of this review will be used to develop a conceptual and theoretical framework to identify the important domains and indicators for appropriate SN involvement initiatives in mental healthcare. However, the methodological variation in approaches used to study SNs might warrant caution when interpreting the findings. The overall value and credibility of this umbrella review will be dependent on the amount, quality, and comprehensiveness of the information available in the primary studies as well as on the evaluation methods used across included reviews.
