# Supplementary material for: Social network interventions in mental healthcare: a protocol for an umbrella review
Source: BMJ Open. 2021 Dec 17;11(12):e052831. doi: 10.1136/bmjopen-2021-052831 (PMC8685933; doi:10.1136/bmjopen-2021-052831)
Supplement: Supplementary data [file bmjopen-2021-052831supp001.pdf]

Appendix 1: Search strategy for Ovid MEDLINE.

1 exp Interpersonal Relations/

2 exp Social Support/

3 exp Social Networking/

4 Community Networks/

5 (social OR communit* OR famil* OR peer* OR friend*) adj3 (network* OR support*) mp. [mp=title, abstract, original title, name of substance word, subject heading word, keyword heading word, protocol supplementary concept, rare disease supplementary concept, unique identifier]

6 1 OR 2 OR 3 OR 4 OR 5

7 exp Mental Disorders/

8 Mental Health/

9 Mentally Ill Persons/

10 ((mental* OR psychiatr* OR psycholog*) adj2 (problem* OR difficult* OR disorder* OR disease* OR ill* OR health*)) mp. [mp=title, abstract, original title, name of substance word, subject heading word, keyword heading word, protocol supplementary concept, rare disease supplementary concept, unique identifier]

11 7 OR 8 OR 9 OR 10

12 exp Mood Disorders/ OR Depressive Disorder/ OR Bipolar Disorder/ OR affective disorder* OR depressive disorder* OR depression* OR mania* OR bipolar disorder* OR dysthymic disorder* OR dysthymia* OR affective disturbance* OR affective ill* OR mood disturbance*mp. [mp=title, abstract, original title, name of substance word, subject heading word, keyword heading word, protocol supplementary concept, rare disease supplementary concept, unique identifier]

13 exp Anxiety Disorders/ OR Neurotic Disorders/ OR Obsessive-Compulsive Disorder/ OR Panic Disorder/ OR Phobic Disorders/ OR Stress Disorders, Post-traumatic/ OR anxiety disorder* OR neurotic disorder* OR obsessive-compulsive disorder* OR panic disorder* OR phobic disorder* OR phobia* OR generalized anxiety disorder* OR generalised anxiety disorder* OR posttraumatic stress disorder* mp. [mp=title, abstract, original title, name of substance word, subject heading word, keyword heading word, protocol supplementary concept, rare disease supplementary concept, unique identifier]

14 exp "Trauma and Stressor Related Disorders"/ OR Stress Disorders, Traumatic/ OR Psychological Trauma/ OR Psychological Distress/ OR Stress, Psychological/ OR

trauma* OR stress disorder* OR psychological distress* OR emotional distress* mp. [mp=title, abstract, original title, name of substance word, subject heading word, keyword heading word, protocol supplementary concept, rare disease supplementary concept, unique identifier]

15 exp Personality Disorders/ OR personality disorder* OR personality patholog* OR personality difficult* OR disordered personalit* mp. [mp=title, abstract, original title, name of substance word, subject heading word, keyword heading word, protocol supplementary concept, rare disease supplementary concept, unique identifier]

16 exp Substance-Related Disorders/ OR Alcohol-Related Disorders/ OR Illicit Drugs/ OR Alcoholism/ OR Binge Drinking/ OR "drug abuse" OR "substance abuse" OR "alcohol abuse" OR "drug dependence" OR "substance dependence" OR "alcohol dependence" OR "drug addiction" OR "substance addiction" OR "alcohol addiction" OR "substance-use disorder" OR "alcohol-use disorder" OR alcoholi* OR binge drink* mp. [mp=title, abstract, original title, name of substance word, subject heading word, keyword heading word, protocol supplementary concept, rare disease supplementary concept, unique identifier]

17 exp Affective Disorders, Psychotic/ OR Psychotic Disorders/ OR Paranoid Disorders/ OR Schizophrenia/ OR delusion* OR hallucinat* OR schizophren* OR "psychosis" OR "schizoaffective" OR "psychotic" OR "paranoid"

18 exp "Feeding and Eating Disorders"/

19 ((exp Anorexia Nervosa/ OR Anorexia/) OR (exp Bulimia Nervosa/ OR Bulimia/ OR Binge-Eating Disorder/)) OR ((anorexi* OR bulimi*) AND nervosa) OR eating disorder* OR binge-eat* OR (bing* adj2 eat*) OR (compulsive adj2 (eat* OR vomit* or purg*)) mp. [mp=title, abstract, original title, name of substance word, subject heading word, keyword heading word, protocol supplementary concept, rare disease supplementary concept, unique identifier]

20 12 OR 13 OR 14 OR 15 OR 16 OR 17 OR 18 OR 19

21 ((systematic OR scoping OR literature) ADJ1 (review* OR overview*)) OR "review* of reviews" OR meta-analy* OR metaanaly* OR ((systematic OR evidence) ADJ1 assess*) OR metasynthe* OR meta-synthe*.tw. OR exp Review Literature as Topic/ OR exp Review/ OR Meta-Analysis as Topic/ OR Meta-Analysis/ OR "systematic review"/

22 6 AND 11 AND 20 AND 21

23 Limit 22 to (English and yr= “2010-2020)
